# Complete Genome Sequence of *Mycobacterium chimaera* Strain AH16

**DOI:** 10.1128/genomeA.01276-16

**Published:** 2016-11-23

**Authors:** Nabeeh A. Hasan, Jennifer R. Honda, Rebecca M. Davidson, L. Elaine Epperson, Matthew J. Bankowski, Edward D. Chan, Michael Strong

**Affiliations:** a Center for Genes, Environment and Health, National Jewish Health, Denver, Colorado, USA; b Department of Medicine, National Jewish Health, Denver, Colorado, USA; c Division of Pulmonary Sciences and Critical Care Medicine, University of Colorado Denver, Anschutz Medical Campus, Aurora, Colorado, USA; d Computational Bioscience Program, University of Colorado Denver, Anschutz Medical Campus, Aurora, Colorado, USA; e Diagnostic Laboratory Services Inc., Aiea, Hawaii, USA; f Departments of Pathology and Tropical Medicine, Medical Microbiology and Pharmacology, John A. Burns School of Medicine, University of Hawai’i at Manoa, Honolulu, Hawaii, USA; g Denver Veterans Affairs Medical Center, Denver, Colorado, USA

## Abstract

*Mycobacterium chimaera* is a nontuberculous mycobacterial species that causes cardiovascular, pulmonary, and postsurgical infections. Here, we report the first complete genome sequence of *M. chimaera*. This genome is 6.33 Mbp, with a G+C content of 67.56%, and encodes 4,926 protein-coding genes, as well as 74 tRNAs, one ncRNA, and three rRNA genes.

## GENOME ANNOUNCEMENT

*Mycobacterium chimaera* is a nontuberculous mycobacterial species within the *Mycobacterium avium* complex (MAC). The use of single genes such as the 16s rRNA or *rpo*B for species identification often misidentifies *M. chimaera* as the closely related MAC species *M. intracellulare* (1, 2). *M. chimaera* is an opportunistic environmental pathogen implicated in cardiovascular and pulmonary infections (1, 3). In health-care settings, the occurrence and spread of *M. chimaera* infections have increased in prevalence in recent years, including recent outbreaks that have been attributed to contaminated medical equipment (4–6). Despite the public health relevance of *M. chimaera*, only three draft genomes are publicly available, none of which is a complete genome (7). To improve our understanding of this emerging pathogen, we sequenced and report here the first complete genome of *M. chimaera*. 

*M. chimaera* strain AH16, isolated from an 87-year-old male with suspected mycobacterial lung disease in O’ahu, Hawai’i, was identified using partial *rpoB* sequencing. The genome of *M. chimaera* AH16 was sequenced using Pacific Biosciences (PacBio) RSII single-molecule real-time (SMRT) technology. PacBio sequencing produced 243,370 reads (mean: 6,808 bp; min: 50 bp; max: 45,065 bp; ~262× genome coverage). The *de novo* genome assembly was performed using the Hierarchical Genome Assembly Process (8). The assembly was compared to other MAC genomes using Mauve version 2.3.1 (9). Genomic features were identified and annotated using the NCBI Prokaryotic Genome Annotation Pipeline (10).

The genome of *M. chimaera* AH16 consists of 4 scaffolds equaling 6,328,534 bp (5,851,561-bp chromosome; 437,456-bp plasmid; 25,007-bp plasmid; 14,510-bp plasmid) and a G+C content of 67.56%. A total of 4,926 coding sequences (CDSs) were predicted, including 3,161 CDSs (64.17%) with functional annotations and 1,765 CDSs (35.83%) annotated as hypothetical proteins. Our genome assembly contains 74 tRNAs, one ncRNA, and one rRNA cistron consisting of the 16S, 23S, and 5S rRNA genes.

Whole-genome alignments of *M. chimaera* AH16 and six MAC genomes revealed 511,189 variable single nucleotide polymorphisms (SNPs), including: 459,672 SNPs compared to *M. avium* subsp. *hominis suis* TH135; 64,709 SNPs compared to *M. intracellulare* ATCC 13950; 50,875 SNPs compared to *Mycobacterium* sp. MOTT 36Y; 49,805 SNPs compared to *M. yongonense* 05-0390^T^; and 49,524 SNPs compared to *Mycobacterium* sp. H4Y. Comparing the *M. chimaera* AH16 genome against the *M. avium* subsp. *hominis suis* TH135, *M. intracellulare* ATCC 13950, *M. yongonense* 05-0390^T^, *Mycobacterium* sp. MOTT 36Y, and *Mycobacterium* sp. H4Y genomes, the average nucleotide identities (ANIs) were 85.55%, 96.87%, 97.14%, 97.16%, and 97.22%, respectively. The limited SNP variation (0.78 to 7.26% of the *M. chimaera* AH16 genome) and ANI values observed between *M. chimaera* AH16 and MAC species suggests that the non–*M. avium* MAC species have limited genomic variation. Furthermore, intraspecies genome comparisons of *M. chimaera* AH16 to *M. chimaera* MCIMRL6, MCIMRL4, and MCIMRL2 (accession nos. LJHN00000000, LJHM00000000, and LJHL00000000) have 99.14%, 99.11%, and 99.18% ANIs, respectively, which are within the 95 to 96% cutoff for species boundaries (11).

### Accession number(s).

The genome sequence of *M. chimaera* AH16 is deposited in NCBI GenBank under the accession numbers CP012885 to CP012888.

## References

[1] Wallace RJ, Iakhiaeva E, Williams MD, Brown-Elliott BA, Vasireddy S, Vasireddy R, Lande L, Peterson DD, Sawicki J, Kwait R, Tichenor WS, Turenne C, Falkinham JO 2013 Absence of *Mycobacterium intracellulare* and presence of *Mycobacterium chimaera* in household water and biofilm samples of patients in the United States with *Mycobacterium avium* complex respiratory disease. J Clin Microbiol 51:1747–1752. doi:10.1128/JCM.00186-13. 23536397 PMC3716115

[2] Schweickert B, Goldenberg O, Richter E, Göbel UB, Petrich A, Buchholz P, Moter A 2008 Occurrence and clinical relevance of *Mycobacterium chimaera* sp. nov., Germany. Emerg Infect Dis 14:1443–1446. doi:10.3201/eid1409.071032. 18760016 PMC2603105

[3] Tortoli E, Rindi L, Garcia MJ, Chiaradonna P, Dei R, Garzelli C, Kroppenstedt RM, Lari N, Mattei R, Mariottini A, Mazzarelli G, Murcia MI, Nanetti A, Piccoli P, Scarparo C 2004 Proposal to elevate the genetic variant MAC-A, included in the *Mycobacterium avium* complex, to species rank as *Mycobacterium chimaera* sp. nov. Int J Syst Evol Microbiol 54:1277–1285. doi:10.1099/ijs.0.02777-0. 15280303

[4] Sommerstein R, Rüegg C, Kohler P, Bloemberg G, Kuster SP, Sax H 2016 Transmission of *Mycobacterium chimaera* from heater-cooler units during cardiac surgery despite an ultraclean air ventilation system. Emerg Infect Dis 22:1008–1013. doi:10.3201/eid2206.160045. 27070958 PMC4880077

[5] Kohler P, Kuster SP, Bloemberg G, Schulthess B, Frank M, Tanner FC, Rössle M, Böni C, Falk V, Wilhelm MJ, Sommerstein R, Achermann Y, ten Oever J, Debast SB, Wolfhagen MJ, Brandon Bravo Bruinsma GJ, Vos MC, Bogers A, Serr A, Beyersdorf F, Sax H, Böttger EC, Weber R, van Ingen J, Wagner D, Hasse B 2015 Healthcare-associated prosthetic heart valve, aortic vascular graft, and disseminated *Mycobacterium chimaera* infections subsequent to open heart surgery. Eur Heart J 36:2745–2753. doi:10.1093/eurheartj/ehv342. 26188001

[6] Sax H, Bloemberg G, Hasse B, Sommerstein R, Kohler P, Achermann Y, Rössle M, Falk V, Kuster SP, Böttger EC, Weber R 2015 Prolonged outbreak of *Mycobacterium chimaera* infection after open-chest heart surgery. Clin Infect Dis 61:67–75 doi:10.1093/cid/civ198. 25761866

[7] Mac Aogáin M, Roycroft E, Raftery P, Mok S, Fitzgibbon M, Rogers TR 2015 Draft genome sequences of three *Mycobacterium chimaera* respiratory isolates. Genome Announc 3(6):e01409-15. doi:10.1128/genomeA.01409-15. 26634757 PMC4669398

[8] Chin C-S, Alexander DH, Marks P, Klammer AA, Drake J, Heiner C, Clum A, Copeland A, Huddleston J, Eichler EE, Turner SW, Korlach J 2013 Nonhybrid, finished microbial genome assemblies from long-read SMRT sequencing data. Nat Methods 10:563–569. doi:10.1038/nmeth.2474. 23644548

[9] Darling AE, Mau B, Perna NT 2010 progressiveMauve: multiple genome alignment with gene gain, loss and rearrangement. PLoS One 5:e11147. doi:10.1371/journal.pone.0011147. 20593022 PMC2892488

[10] Tatusova T, DiCuccio M, Badretdin A, Chetvernin V, Ciufo S, Li W 2013 Prokaryotic genome annotation pipeline. In The NCBI handbook, 2nd ed. NCBI, Bethesda, MD. http://www.ncbi.nlm.nih.gov/books/NBK174280.

[11] Richter M, Rosselló-Móra R 2009 Shifting the genomic gold standard for the prokaryotic species definition. Proc Natl Acad Sci U S A 106:19126–19131. doi:10.1073/pnas.0906412106. 19855009 PMC2776425

